# State impulsivity and substance use: A systematic review and meta-analysis protocol

**DOI:** 10.1371/journal.pone.0346779

**Published:** 2026-04-07

**Authors:** Ashmita Mazumder, Suzanne Erb, Marc A. Fournier

**Affiliations:** Department of Psychology, University of Toronto Scarborough, Scarborough, Ontario, Canada; Griffith University, AUSTRALIA

## Abstract

The association between substance use and impulsivity has been documented extensively in the literature. More recently, there has been a shift from viewing impulsivity solely as a stable trait toward examining its moment-to-moment expression in everyday life and how these fluctuations influence substance use. Despite this growing interest, there has not yet been a comprehensive meta-analysis synthesizing findings across studies. Existing reviews have largely focused on trait-level impulsivity, which limits our understanding of how impulsivity operates across real-world contexts. The aim of this proposed meta-analysis is to integrate these findings and quantify the strength of the association between everyday impulsivity and substance use, specifically alcohol, cannabis, and tobacco use. In addition, we aim to identify key moderators of this relationship. For example, we will evaluate whether the strength of association differs by substance (e.g., alcohol vs. cannabis vs. tobacco), by sample characteristics (substance-dependent vs. community), and by methodological factors. We will search major databases (e.g., PsycINFO, PubMed, Web of Science) for peer-reviewed studies as well as unpublished studies reporting associations between everyday impulsivity and substance use. Random-effects models will estimate pooled effect sizes, and subgroup/meta-regression analyses will test moderators. Overall, this proposed meta-analysis aims to provide a comprehensive estimate of the association between everyday impulsivity and alcohol, cannabis, and tobacco use.

## Introduction

Substance use remains one of the most pressing public health issues worldwide, contributing to significant individual and societal costs. Among the most widely used substances are alcohol, cannabis, and tobacco [1]. Regular exposure to these substances has been consistently linked to adverse outcomes, including behavioral impairments such as deficits in memory [2] and motor coordination [3], as well as psychological difficulties such as heightened anxiety [4] and depression [5]. Increasingly, substance use is understood not only as a function of stable individual differences, but also as a dynamic process influenced by momentary psychological states that fluctuate in everyday life. One such state is impulsivity, which may increase vulnerability to substance use. The present meta-analysis aims to synthesize evidence linking self-reported momentary impulsivity to real-time use of alcohol, cannabis, and tobacco.

According to the 2024 National Survey on Drug Use and Health, 46.6% of individuals aged 12 or older reported past-month alcohol use [6], a pattern mirrored in Canada where 78% of those aged 15 + reported past-year consumption [7]. Tobacco/nicotine and cannabis products remain highly prevalent as well, with 16.7% and 15.4% of the population, respectively, reporting use in the past month [6]. Cannabis has also remained the most used drug in Canada with 21% of the population reporting its use [7]. By comparison, population-level prevalence of non-cannabis illicit substances (such as heroin, opioids, and methamphetamine) remains substantially lower, with past-year use typically reported by approximately 2–3% of individuals [6]. Importantly, lower prevalence does not imply lower harm; rather, these differences highlight the relative ubiquity of alcohol, tobacco, and cannabis in the general population. Beyond prevalence, the use of non-cannabis illicit substances is often characterized by illegality, stigma, and limited availability, which strongly shape behavior and overshadow everyday decision-making [8]. In contrast, alcohol, cannabis, and tobacco use are reinforced by cultural and social norms [9,10], positive beliefs [11,12], belonging [13], and perceptions of socially acceptability [14]. These factors, combined with their high accessibility, make them especially important targets for the present review.

One personality trait often implicated in substance use, as both a cause and a consequence, is impulsivity [15–17]. Broadly defined as behavior without forethought, impulsivity is considered a core feature of many clinical disorders, such as borderline personality disorder, bipolar disorder, and schizophrenia [18,19]. Moreover, in many cases, impulsivity is associated with some of the more severe consequences of such disorders, such as suicide attempts, aggressive behavior, and self-harm [20,21]. The link between impulsivity and substance use is well established and supported by both longitudinal and cross-sectional evidence [22–25]. The association is also remarkably robust, persisting across different study designs, populations, and measurement approaches. For example, urgency and sensation-seeking facets of impulsivity predict alcohol use even after accounting for contextual factors such as peer and parental drinking, underscoring its unique role beyond environmental influences [26]. Furthermore, impulsivity mediates the relationship between early childhood adversity and substance use, highlighting its role as a developmental pathway to risk [27].

While traditionally studied as a trait-level characteristic, recent research emphasizes state-level fluctuations in impulsivity and their relevance for clinical outcomes, including substance use [17]. This shift has been facilitated by methodological advances, including the development of the experience sampling method (ESM), which allow researchers to capture real-time variability in psychological states, maximize ecological validity, and minimize recall bias [28]. The method includes daily diary studies, where participants report on their experiences once a day, and ecological momentary assessment (EMA), where data are collected multiple times a day.

By providing repeated, momentary snapshots of individuals’ daily lives, ESM allows researchers to move beyond trait-level associations and identify the dynamic processes that underlie behavior. Past research has found associations between state impulsivity and passive suicidal ideation [29], momentary posttraumatic stress disorder symptoms [30], and binge-eating [31] among other clinical outcomes. Similarly, these intensive longitudinal approaches can reveal how momentary shifts in impulsivity contribute to substance use patterns across the day, or how fluctuations in psychological states interact with situational contexts to predict use in real time. Indeed, state impulsivity does seem to influence alcohol, cannabis, and tobacco use by incrementally predicting substance use behaviors and adverse outcomes over and above trait impulsivity [32–36]. Overall, this underscores the importance of assessing the role and influence of state impulsivity in everyday substance use.

Consistent with this primary literature, prior reviews and meta-analyses demonstrate moderate and reliable associations between trait impulsivity and both alcohol use and alcohol-related problems, with urgency-related traits showing particularly strong links to problematic drinking [37–39]. Similarly, smokers exhibit elevated levels of trait impulsivity relative to non-smokers [40], and impulsivity is associated to cannabis use and negative cannabis-related consequences [41,42]. Although impulsivity is also elevated among users of non-cannabis illicit substances, research tends to find most consistent associations between impulsivity and stimulants [43,44]. In contrast, opioid use is often related to impaired value-based decision-making, but motor response inhibition deficits appear to be smaller and less consistent [45]. Similarly, evidence related to hallucinogens are inconsistent with some studies showing non-significant association with impulsivity [46]. While impulsivity is elevated among those that use non-cannabis illicit substances, research on these drugs often relies on behavioral tasks that operationalize state impulsivity in laboratory or treatment settings. These tasks often show weak convergence with self-reported impulsivity and yield highly heterogeneous findings [47,48]. In contrast, the evidence base for alcohol, cannabis, and tobacco is more extensive and methodologically diverse, making these substances especially relevant for examining the dynamic role of impulsivity in everyday life [48,49].

### The current review

As reviews to date have primarily addressed cross-sectional, longitudinal, or behavioral associations between trait impulsivity and substance use, there is a clear need for a focused review examining state-level impulsivity in relation to alcohol, cannabis, and tobacco use in non-clinical populations. This would advance our understanding of how momentary impulsivity processes unfold in daily life beyond stable trait vulnerabilities. To date, the evidence remains fragmented across individual studies and a systematic synthesis is needed to clarify the strength and consistency of associations between state impulsivity and substance use, and to determine whether it varies by important factors such as the type of substance examined (e.g., alcohol, cannabis, tobacco) or sample characteristics (e.g., community versus substance-dependent populations). Addressing these questions will establish a clearer foundation for understanding how momentary fluctuations in impulsivity contribute to substance use and may ultimately inform prevention and intervention strategies that are sensitive to real-time risk processes.

Guided by the PICOS (Participants, Intervention/Exposure, Comparison, Outcome, Study design) [50] framework, the objective of this systematic review and meta-analysis is to synthesize and quantitatively evaluate evidence on the between- and within-person association of state impulsivity to everyday substance use. The population of interest includes human participants drawn from both non-clinical (e.g., community and student samples) and substance-dependent samples. The present review excludes studies solely conducted on clinical populations as they are characterized by chronic deficits in inhibitory control and emotion regulation that manifest as persistently high trait impulsivity [51,52]. In such populations, elevations in impulsivity often reflect enduring neurocognitive dysfunction, including structural and functional alterations in frontal control systems, rather than momentary fluctuations in self-regulatory capacity [53]. For example, neurological conditions such as traumatic brain injury are linked to lasting disinhibition and impaired impulse control resulting from damage to frontal systems [54]. These neurobiologically embedded impulsivity profiles differ qualitatively from the transient, context-sensitive impulsivity observed in the general population. As such, including exclusively clinical or neurological samples risks conflating trait-based pathology with the state-level impulsivity processes that are the primary focus of this review.

State impulsivity is broadly construed to encompass related constructs such as momentary self-control and self-regulation, assessed repeatedly over time. This inclusive definition reflects theoretical perspectives that conceptualize impulsivity as the momentary failure or fluctuation of self-regulatory processes, as well as the diversity of operationalizations used in experience sampling research. Accordingly, studies assessing conceptually overlapping state-level constructs (e.g., momentary inhibitory control, regulation, or perceived self-control) will be considered eligible, provided they capture within-person variability over time. Comparisons will be made within individuals, consistent with ecological momentary assessment and daily diary methodologies. The primary outcomes of interest will be state-level use of alcohol, cannabis, and tobacco. Eligible study designs will include observational studies employing methods capable of estimating within-person associations.

Beyond establishing the general relationships between self-reported state impulsivity and alcohol, cannabis, and tobacco use, we also seek to examine several key moderators. First, we will consider sample characteristics, testing whether associations differ between substance-dependent and community populations. Second, we will evaluate whether the type of substance matters, by comparing associations across alcohol, cannabis, and tobacco use. Finally, we will assess whether study design plays a role, contrasting results from EMA studies with those from daily diary studies. Taken together, it is expected that an examination these variables will provide a more nuanced understanding of how state impulsivity relates to substance use across different populations, substances, and methodological approaches. To our knowledge, no prior meta-analysis has focused specifically on state-level impulsivity in relation to real-time substance use, making this review a timely and important contribution to both research and intervention design.

## Methods

We report the protocol and methods for this meta-analysis in accordance with the Preferred Reporting Items for Systematic Reviews and Meta-Analyses Protocols (PRISMA-P) guidelines [S1 File; 55]. The aims and hypotheses of this protocol are registered on OSF (https://osf.io/rp23a/?view_only=f5ecd8b078574a1595a7e67878e04a9a). Upon completion of the systematic review and meta-analysis, the extracted data, analytic code, and all subsequent updates will be made publicly available on the same OSF page. The final meta-analysis will be reported following the PRISMA 2020 guidelines [56].

### Eligibility criteria

Eligibility criteria are outlined below based on the PICOS [50] approach.

#### Participants.

We will include studies that examine human participants drawn from both non-clinical and substance-dependent samples. Eligible non-clinical samples will include individuals from the general population, student groups, and community. We will also consider studies that focus specifically on participants with substance dependence, given their relevance to patterns of use and impulsivity. In contrast, studies will be excluded if they recruited participants with diagnosed clinical or neurological conditions (e.g., ADHD, borderline personality disorder, traumatic brain injury). As noted in the introduction, in these populations, impulsive behavior may reflect enduring neurocognitive dysfunction, medication effects, or illness severity, making it difficult to disentangle momentary, situational changes in impulsivity from stable clinical vulnerability. We acknowledge that this decision limits generalizability to clinical populations; however, it was necessary to isolate momentary impulsivity processes and provide a coherent theoretical foundation for future work extending these models to clinical contexts.

#### Intervention/Exposure.

To be eligible for inclusion, studies will be required to assess both impulsivity and substance use at the state or momentary level, capturing within-person fluctuations over time. This focus will allow us to synthesize evidence on dynamic, real-time processes rather than trait-level tendencies or baseline indicators. The exposure of interest is state-level impulsivity, broadly construed to include closely related constructs such as momentary self-control or self-regulation, reflecting fluctuations in self-regulatory capacity. We will exclude studies that measure only trait impulsivity or baseline substance use, as well as those that operationalize state impulsivity exclusively through risky behaviors such as gambling.

#### Comparison.

No explicit control or comparison group is required for study eligibility. Given the use of ESM, eligible studies must examine within-person associations between momentary impulsivity and substance use. Studies that additionally include between-group comparisons (e.g., substance-dependent vs non-clinical samples) will be included provided that within-person analyses are reported. At the synthesis stage, and where statistical power permits, we will compare effect estimates across studies examining state impulsivity in relation to alcohol, cannabis, and tobacco use separately, in order to assess whether associations vary as a function of substance type.

#### Outcome.

We will include studies that report state-level alcohol, cannabis, and/or tobacco use, reflecting momentary engagement with these substances in daily life. Studies that examine use of other drugs will be excluded. Studies assessing poly-substance use will be included only if the substances assessed are limited to combinations of alcohol, tobacco, and/or cannabis.

#### Study design.

We will include studies that used ESM designs, as these approaches align with a focus on within-person fluctuations in impulsivity and substance use. Eligible studies must have been conducted in naturalistic settings (i.e., outside a laboratory context). To ensure adequate within-person temporal resolution, studies will be required to include repeated assessments over a minimum monitoring period (e.g., ≥ 24 hours / ≥ 2 days). We will exclude studies that relied solely on cross-sectional designs, as well as review articles, meta-analyses, and case studies.

### Information sources

Studies published in peer-reviewed journals will be included. In addition to published studies, we will attempt to identify relevant unpublished work to reduce potential publication bias. Our search will not be restricted by language, geographic location, or date of publication. Articles not published in English will be screened for relevancy based on their title and abstract. We will use readily available translation tools (e.g., google translate) to facilitate data extraction and evaluation, given that no professional translation resources are available to our team.

### Search strategy

The search strategy was developed by the authors in consultation with a senior liaison librarian. An initial comprehensive search was conducted in August 2025, with an updated search planned after initial data extraction to capture newly published studies before manuscript submission. Any additional articles will undergo title/abstract screening, full-text screening, and data extraction prior to synthesis and writing. No further updates are planned unless a subsequent review is undertaken.

Planned searches for peer-reviewed articles were conducted on: OVID Medline, OVID Embase, OVID PsycInfo, ProQuest PsycInfo, EBSCO CINAHL, Web of Science: Core Collection, PubMed, and Scopus. Studies were obtained through advanced searches in all databases. Queries contained wildcards (use of asterisks), Boolean operators (AND/OR), search operator precedence (use of parentheses), and proximity search, to refine the search process. The strategy combined three core concepts: (1) impulsivity and self-regulation, (2) substance use, and (3) experience sampling methodology. Searches were limited to human studies. No restrictions were placed on publication year. An overview of the search concepts and keyword terms is provided in Table 1. The full search strategies for each database are provided in the Supplementary Materials (S1–S5 Tables).

**Table 1:** Search terms used for PubMed, Web of Science, and Scopus.

Topic	Mesh Headings	Sample Keywords
State / Momentary Impulsivity	Impulsive Behavior; Self-Control; Inhibition, Psychological; Self-Regulation	“impulsive behavior*” OR “impulse control” OR “self-control” OR moment* impuls*” OR “moment* self-control” OR “moment* control” OR “moment* disinhibit*” OR “state impuls*” OR “state self-control” OR OR “state disinhibit*” OR “state inhibit*
Substance Use (Alcohol, Cannabis, Tobacco)	Alcohol Drinking; Alcohol Use Disorder; Cannabis Use; Marijuana Use; Tobacco Use; Smoking; Nicotine; Substance-Related Disorders	“alcohol*” OR “alcohol use” OR “alcoholism” OR “cannabis use” OR “marijuana use” OR “tobacco*” OR “tobacco smoking” OR “nicotine*” OR “smoking*” OR “vaping*” OR “cigarette*” OR “e-cigarette*”
EMA / Daily Diary Methods	Ecological Momentary Assessment	“experience sampling” OR “ambulatory assessment” OR “ecological momentary assessment” OR “EMA” OR “momentary” OR “ESM” OR “daily diary” OR “real-time data” OR “intensive longitudinal”

To identify relevant unpublished work, searches will be conducted in the Open Science Framework (https://osf.io/preprints/discover) across multiple preprint repositories (e.g., PsyArXiv, SocArXiv, EdArXiv) using combinations of the phrases “state impulsivity,” “momentary impulsivity,” “state self-control,” “momentary self-control,” “ecological momentary assessment,” “daily diary,” together with “alcohol,” “cannabis,” or “tobacco.” We will also search the ProQuest Dissertations and Theses database using these terms to identify doctoral or master’s research not yet published in peer-reviewed outlets. Finally, we will conduct supplementary searches in Google Scholar using the same keywords and will screen the first 1,000 results to identify additional unpublished or in-press studies that may not have been captured through database searches.

### Study records

#### Data management and selection process.

Screening will be conducted in Covidence, a web-based platform that streamlines literature reviews by managing duplicate removal, screening, and data extraction. Screening will occur in two stages: title/abstract screening and full-text screening. At each stage, two independent reviewers will assess whether articles meet the inclusion and exclusion criteria outlined above. Exclusion criteria will be consistently applied across both stages to ensure that only eligible studies are advanced to data extraction. Any disagreements between reviewers will be discussed and resolved collaboratively by the study team.

#### Data collection process.

Two independent reviewers will extract data from each included article using a standardized extraction form, as outlined in Table 2. Data extraction will be conducted in Covidence, with each reviewer completing the form independently and without access to the other’s responses until their own extraction is finished. Following this, the two sets of extracted data will be compared. In cases where discrepancies arise, the reviewers will meet to discuss the differences and reach consensus on the most accurate and complete information, combining responses where appropriate.

**Table 2:** Data items.

Study description: lead author, year, country (if available)
Participant characteristics: sample size, mean age (SD), gender (% female), ethnicity (% White ethnicity)
Sample characteristics: community (0) or substance dependent (1)
Substances included alcohol, cannabis (and cannabis products), nicotine/tobacco
Baseline substance use characteristics: mean scores on substance use measures (SD)
State substance use outcomes: endorsement of use, quantity
Alcohol use quantity will be coded if researchers report the number of drinks: beers, glasses of wine, shots, and mixed drinks
Cannabis use quantity will be coded if researchers report the number of marijuana hits and the hours spent high
Tobacco/Nicotine quantity will be coded if researchers report the number of puffs or number of cigarettes
State impulsivity measure used, aggregate scores (between-persons)
Information on predictors (other than impulsivity) measured: construct(s) assessed, measurement type (e.g., self-report, behavioral task)
Study type: observational, interventional, both
ESM delivery mode: mobile phone, smartphone, hand-held device
ESM method: signal-contingent, event-contingent, multiple
ESM design characteristics: number of days, attrition rate, incentive, compliance rate, % of complete prompts
Frequency of ESM assessments: daily (0) or multiple times per day (1)
Preregistration: not preregistered (0) or preregistered (1)
Type of statistical model used: e.g., hierarchical/multilevel model, multilevel structural equation model
Person-mean-centering of time varying predictor: no (0) or yes (1)
Level 2 predictors in the same model: no (0) or yes (1)
Number of observations used for the within-person analysis
Number and type of other within-person psychological or contextual predictor(s) included in model
Whether the within- and between-person effects of the time-varying predictor were simultaneously modeled: no (0) or yes (1)
Coefficients (e.g., r, b), standard errors, t-value, df, p-value for the within- and between-person associations.

### Risk of bias

In the absence of existing quality appraisal tools for ESM studies, we developed a checklist based on existing literature [57], including the “Checklist for Reporting EMA Studies [58].” This form can be found in Table 3. Quality indicators for all papers will be coded by the first author. Each item will be scored on a no/yes (0/1) scale. If we are unable to find the relevant information for each item, then we will score it as 0. Instead of a sum quality score, we will present how each paper scored on each of the items.

**Table 3:** Quality appraisal tool.

1.	Was a rationale for EMA design provided (i.e., for why an EMA design was chosen to examine the research question)?
2.	Were the hypotheses and goals stated clearly?
3.	Were the instruments used shown to be reliable?
4.	Were the characteristics of participants highlighted clearly?
5.	Was the study design clearly described?
6.	Was an a priori power analysis conducted to determine the sample size?
7.	Were design features incorporated to address potential sources of bias (e.g., reactivity) or participant burden (e.g., EMA questions appearing in different orders)?
8.	Did the authors report whether study dropout or non-adherence to EMAs (e.g., missed prompts) were related to specific variables?
9.	Were withdrawals and drop-outs reported in terms of numbers and/or reasons?
10.	Were the statistical tests used to assess the main outcomes appropriate?
11.	Were the main findings of the study clearly described?
12.	Were actual probability values reported (e.g., 0.035 rather than <0.05) for the main outcomes except where the probability value was less than 0.001?

### Data synthesis

#### Qualitative synthesis.

The review will include a narrative synthesis of results in addition to a quantitative synthesis. Studies will be grouped and compared based on key methodological and conceptual characteristics, including the type of substance assessed, daily diary vs. EMA, and community vs. substance-dependant populations. Within each grouping, we will qualitatively summarize the direction, magnitude, and consistency of between- and within-person associations between momentary impulsivity and substance use, highlighting patterns that emerge across studies and identifying areas of convergence or divergence. This narrative synthesis will provide a coherent integration of findings across diverse ESM designs and will contextualize quantitative results by clarifying the associations unfold across substances, populations, and methodological approaches. Importantly, this synthesis will identify methodological limitations and gaps in the existing literature, helping to inform priorities for future research.

#### Quantitative synthesis.

The primary effect size of interest will be Pearson’s *r*, reflecting the strength and direction of the association between state impulsivity and substance use. Relevant coefficients will be converted to *r* using established methods described below. For within-person effects, we will record whether predictors are person-mean centered before extraction. When studies report standardized beta coefficients (*β*) from regression models, we will apply the Peterson & Brown transformation [59]: *r* = *β* + 0.05 × *λ,* where *λ* is typically assumed to be 1 in the absence of other information. The Rosenthal & Rosnow formula [60]: *r* = √(*t*^2^ / (*t*^2^ + df)) method will allow us to derive an *r* effect size from the reported *t*-statistic and degrees of freedom (number of prompts for within-person effects and number of participants for between-person effects), reflecting the partial effect of the predictor while adjusting for covariates.

Considering that some of this information may not be available in some papers, we will additionally calculate effect sizes using the metafor/esc package, based on unstandardized *b* and *p*-value. If any other strategies are used to calculate effect size, we will report that transparently in the final manuscript. Where possible, we will distinguish between within-person and between-person associations. Studies reporting both types of effects will be coded and synthesized separately in subgroup analyses. All analyses will be conducted in R. In cases where the effect size cannot be estimated, we will contact the authors of the paper to request relevant information.

Following recommendations from existing literature [61], we will apply the Fisher’s *z* transformation to all *r* values prior to analysis to address the bias and non-constant variance associated with raw correlations. To further minimize small-sample bias, we will apply the small-sample correction to Fisher’s *z*, defined as: *z*_adj = *z* × (4*n* − 2) / (4*n* − 1) [where *n* = sample size of the study]

Meta-analyses will be conducted using a random-effects model to account for both within- and between-study variability. Heterogeneity and publication bias will be assessed using the Q statistic, τ², funnel plots, and I². To examine potential sources of heterogeneity, moderator analyses will be performed using meta-regression. Specifically, we will test whether the strength of the association between state impulsivity and substance use varies by (1) substance type (alcohol, cannabis, tobacco), (2) sample type (substance-dependent vs. community sample), (3) study design characteristics (EMA vs. daily diary), and (4) the interaction between substance type and sample type. Following commonly used thresholds in prior work [62,63], moderator analyses will only be conducted when at least five effect sizes are available for each cell.

#### Confidence in cumulative evidence.

Due to the nature of this study, measures of cumulative evidence, such as GRADE [64], will not be addressed. Such approaches are typically designed for intervention or clinical outcome studies, where evidence quality is assessed based on domains such as risk of bias, imprecision, and consistency of treatment effects. In contrast, the present review synthesizes observational studies of state-level associations between impulsivity and substance use, making GRADE less appropriate and of limited added value. Instead, we will prioritize methodological transparency, detailed reporting of study characteristics, and moderator analyses to provide clarity on the robustness and generalizability of findings.

## Discussion

This protocol outlines a meta-analysis aimed at quantitatively synthesizing the evidence on state impulsivity and alcohol, cannabis, and tobacco use. Although trait-level impulsivity has been widely studied and consistently linked to substance use, research examining impulsivity as a dynamic, momentary process is fragmented. By systematically identifying, evaluating, and integrating this body of work, the present work aims to provide a clearer understanding of how within-person fluctuations in impulsivity contribute to substance use in daily life.

It is anticipated that the planned synthesis will make several important contributions. First, it will clarify the overall strength of the association between state impulsivity and substance use, helping to determine whether the robust relationships observed at the trait level extend to the state level. Second, it will allow for an evaluation of whether these associations vary by substance type, sample characteristics, or methodological features. Alcohol, cannabis, and tobacco each have distinct patterns of use, social acceptability, and pharmacological effects, and it is possible that the role of state impulsivity varies across these substances. Third, the review will examine how sample characteristics (e.g., community vs. substance-dependent samples) shape the strength of these associations. This will in turn provide insight into whether impulsivity functions as a universal risk factor or whether its influence is contingent on particular populations or levels of use severity. Finally, by synthesizing evidence from different ESM methodologies, the review will highlight the importance of studying processes as they unfold in daily life. Such findings can inform both the refinement of measurement approaches and the development of real-time interventions.

At the same time, several challenges are anticipated. A primary concern is heterogeneity. Studies vary considerably in how state impulsivity is conceptualized and measured, with some specifically focusing on facets such as urgency or sensation seeking and others employing single-item ratings of general impulsivity. This heterogeneity reflects both the diversity of theoretical perspectives on impulsivity and the variability in how momentary self-regulatory processes are operationalized in experience sampling research. Although adopting a broad conceptualization of state impulsivity is necessary to capture the full scope of relevant evidence and avoid excluding theoretically overlapping constructs (e.g., momentary self-control or self-regulation), this approach may contribute to increased variability in effect estimates and limit direct comparability across studies. Similarly, substance use is operationalized in diverse ways, ranging from the number of drinks per occasion to binary indicators of use versus non-use. This variability may complicate synthesis and limit comparability across studies. Another challenge relates to the uneven distribution of research across substances. Whereas alcohol has been studied extensively using EMA methods, cannabis and especially tobacco are represented by fewer studies, which may constrain moderator analyses or reduce statistical power to detect substance-specific effects. A third challenge concerns the predominance of young adult and North American samples, raising questions about the generalizability of findings to other age groups, cultural contexts, or clinical populations.

Another limitation of the present review concerns the assessment of study quality. Although a recently developed quality appraisal tool for experience sampling methods (ESM-Q) [65] has been proposed, we will not apply this tool here. The ESM-Q relies heavily on item-level and protocol-specific information (e.g., prompt wording and response options), which may be inconsistently reported, subjectively analyzed, or unavailable across many eligible studies. Given the heterogeneity of ESM designs and reporting practices, applying the ESM-Q would have resulted in substantial missing data and potentially biased quality ratings. Instead, we will employ a more flexible risk-of-bias approach suitable for heterogeneous observational ESM studies, while acknowledging the need for improved and standardized reporting to facilitate more granular quality appraisal in future research. Finally, although our search is not limited by language, we relied on AI-based translation tools to evaluate articles not published in English, which may introduce bias. However, this approach will allow us to include a broader range of relevant studies that might otherwise be excluded due to language constraints. We will highlight these gaps explicitly, interpret findings cautiously, and recommend future research to address underrepresented substances and substance combinations. In addition, we will note the demographic limitations of the literature in our discussion and encourage future work with more generalizable populations.

In sum, this protocol outlines an approach to synthesizing emerging literature on impulsivity and substance use in daily life. By integrating evidence across alcohol, cannabis, and tobacco, the review will provide a more comprehensive understanding of how momentary fluctuations in impulsivity shape real-world patterns of use.

## Supporting information

S1 File PRISMA-P 2015 Checklist. PRISMA-P (Preferred Reporting Items for Systematic review and Meta-Analysis Protocols) 2015 checklist: Recommended items to address in a systematic review protocol.
(DOCX)

S1 Table Search terms used for PubMed, Web of Science, and Scopus. This table presents the complete Boolean search strings used to identify relevant studies in PubMed, Web of Science Core Collection, and Scopus.
(DOCX)

S2 Table Search terms used for PsycINFO. This table presents the complete Boolean search strings used to identify relevant studies in PsycINFO.
(DOCX)

S3 Table Search terms used for EBSCO CINAHL. This table presents the complete Boolean search strings used to identify relevant studies in EBSCO Cumulative Index to Nursing and Allied Health Literature.
(DOCX)

S4 Table Search terms used for OVID Medline and OVID Embase. This table presents the complete Boolean search strings used to identify relevant studies in OVID Embase and OVID Medline.
(DOCX)

S5 Table Search terms used for OVID PsycINFO. This table presents the complete Boolean search strings used to identify relevant studies in OVID PsycINFO.
(DOCX)
