# Supplementary material for: State impulsivity and substance use: A systematic review and meta-analysis protocol
Source: PLoS One. 2026 Apr 7;21(4):e0346779. doi: 10.1371/journal.pone.0346779 (PMC13056172; doi:10.1371/journal.pone.0346779)
Supplement: S1 Table — This table presents the complete Boolean search strings used to identify relevant studies in PubMed, Web of Science Core Collection, and Scopus. (DOCX) [file pone.0346779.s002.docx]

**Table S1: Search terms used for PubMed, Web of Science, and Scopus.**

| PubMed (NIH Library of Medicine)/ Web of Science: Core Collection/ Scopus (Elsevier) | ("substance use" OR "substance" OR "substance abuse" OR "alcohol*" OR "drink*" OR "binge drinking" OR "heavy drinking" OR “alcohol use” OR "alcoholism" OR "alcohol intoxication" OR "alcoholic intoxication" OR “alcohol consumption” OR "cannabi*" OR "marijuana" OR "weed" OR "THC" OR "pot" OR "cannabis use" OR "marijuana use" OR "tobacco*" OR "tobacco smoking" OR "nicotine*" OR "smoking*" OR "vaping*" OR "cigarette*" OR "e-cigarette*" OR "e-cig*" OR "JUUL*" OR "hookah" OR "pipe smoking" OR "smokeless tobacco" OR "nicotine abuse*" OR "nicotine addict*" OR "nicotine dependen*" OR "nicotine disorder*" OR "nicotine misuse" OR "tobacco abuse*" OR "tobacco addict*" OR "tobacco dependen*" OR "tobacco disorder*" OR "tobacco misuse" OR "cannabinoid abuse*" OR "cannabinoid addict*" OR "cannabinoid dependen*" OR "cannabinoid disorder*" OR "cannabinoid misuse" OR "cannabis abuse*" OR "cannabis addict*" OR "cannabis dependen*" OR "cannabis disorder*" OR "cannabis misuse" OR "alcohol abuse*" OR "alcohol addict*" OR "alcohol dependen*" OR "alcohol disorder*" OR "alcohol misuse" OR "marijuana smoking") AND ("experience sampling" OR "ambulatory assessment" OR "ecological momentary assessment"  OR "EMA" OR "momentary" OR "ESM" OR "daily diary" OR "real-time data" OR "intensive longitudinal" OR "in-the-moment" OR "mobile assessment" OR "smartphone assessment" OR "event-contingent") AND (impulsiv* OR impuls* OR "self-control" OR "self control" OR disinhibit* OR inhibit* OR "state impulsivity" OR "momentary impulsivity"  OR "impulsive behavior*" OR "impulsive behaviour*" OR "impulsive decision*" OR "impulse control" OR "impulse control disorder" OR "conduct disorder" OR "self-regulation" OR "choice behavior" OR "choice behaviour" OR "behavior control" OR "behaviour control" OR "behavioral inhibition system" OR "behavioural inhibition system" OR "behavioral inhibition" OR "behavioural inhibition" OR "response inhibition"  OR "lack of control" OR "poor inhibition" OR "inhibitory failure" OR urgency OR "positive urgency" OR "negative urgency" OR premeditation OR "lack of premeditation" OR non-planning OR "lack of planning"  OR perseverance OR "lack of perseverance" OR "delay discounting" OR "impulsive choice" OR "impulsive action" OR "gambling" OR "sensation seeking" OR "sensation-seeking" OR "daily impuls*" OR "daily self-control" OR "daily control" OR "daily disinhibit*" OR "daily inhibit*" OR "moment* impuls*" OR "moment* self-control" OR "moment* control" OR "moment* disinhibit*" OR "moment* inhibit*" OR "state impuls*" OR "state self-control" OR "state control" OR "state disinhibit*" OR "state inhibit*"))) |
| --- | --- |

This table presents the complete Boolean search strings used to identify relevant studies in PubMed, Web of Science Core Collection, and Scopus.
