# Supplementary material for: State impulsivity and substance use: A systematic review and meta-analysis protocol
Source: PLoS One. 2026 Apr 7;21(4):e0346779. doi: 10.1371/journal.pone.0346779 (PMC13056172; doi:10.1371/journal.pone.0346779)
Supplement: S2 Table — This table presents the complete Boolean search strings used to identify relevant studies in PsycINFO. (DOCX) [file pone.0346779.s003.docx]

**Table S2: Search terms used for PsycINFO.**

| PsycINFO (ProQuest) | ("substance use" OR "substance" OR "substance abuse" OR "alcohol*" OR "drink*" OR "binge drinking" OR "heavy drinking" OR "alcohol use" OR "alcoholism" OR "alcohol intoxication" OR "alcoholic intoxication" OR "alcohol consumption" OR "cannabi*" OR "marijuana" OR "mari#uana" OR "weed" OR "THC" OR "pot" OR "cannabis use" OR "marijuana use" OR "tobacco*" OR "tobacco smoking" OR "nicotine*" OR "smoking*" OR "vaping*" OR "cigarette*" OR "e-cigarette*" OR "e-cig*" OR "JUUL*" OR "hookah" OR "pipe smoking" OR "smokeless tobacco" OR (nicotine NEAR/2 (abuse* OR addict* OR dependen* OR disorder* OR misuse)) OR (tobacco* NEAR/2 (abuse* OR addict* OR dependen* OR disorder* OR misuse)) OR (cannabinoid* NEAR/2 (abuse* OR addict* OR dependen* OR disorder* OR misuse)) OR (cannabis NEAR/2 (abuse* OR addict* OR dependen* OR disorder* OR misuse)) OR (mari#uana* NEAR/2 (abuse* OR addict* OR dependen* OR disorder* OR misuse)) OR (alcohol* NEAR/2 (abuse* OR addict* OR dependen* OR disorder* OR misuse)) " OR " marijuana smoking "") AND ("experience sampling" OR "ambulatory assessment" OR "ecological momentary assessment" OR "EMA" OR "momentary" OR "ESM" OR "daily diary" OR "real-time data" OR "intensive longitudinal" OR "in-the-moment" OR "mobile assessment" OR "smartphone assessment" OR "event-contingent") AND (impulsiv* OR impuls* OR "self-control" OR "self control" OR disinhibit* OR inhibit* OR "state impulsivity" OR "momentary impulsivity" OR "impulsive behavior*" OR "impulsive behaviour*" OR "impulsive decision*" OR "impulse control" OR "impulse control disorder" OR "conduct disorder" OR "self-regulation" OR "choice behavior" OR "choice behaviour" OR "behavior control" OR "behaviour control" OR "behavioral inhibition system" OR "behavioural inhibition system" OR "behavioral inhibition" OR "behavioural inhibition" OR "response inhibition" OR "lack of control" OR "poor inhibition" OR "inhibitory failure" OR urgency OR "positive urgency" OR "negative urgency" OR premeditation OR "lack of premeditation" OR non-planning OR "lack of planning" OR perseverance OR "lack of perseverance" OR "delay discounting" OR "impulsive choice" OR "impulsive action" OR "gambling" OR "sensation seeking" OR "sensation-seeking " OR (daily NEAR/2 (impuls* OR self-control OR control OR disinhibit* OR inhibit*)) OR (moment* NEAR/2 (impuls* OR self-control OR control OR disinhibit* OR inhibit*)) OR (state NEAR/2 (impuls* OR self-control OR control OR disinhibit* OR inhibit*))) |
| --- | --- |

This table presents the complete Boolean search strings used to identify relevant studies in PsycINFO.
