# Supplementary material for: State impulsivity and substance use: A systematic review and meta-analysis protocol
Source: PLoS One. 2026 Apr 7;21(4):e0346779. doi: 10.1371/journal.pone.0346779 (PMC13056172; doi:10.1371/journal.pone.0346779)
Supplement: S4 Table — This table presents the complete Boolean search strings used to identify relevant studies in OVID Embase and OVID Medline. (DOCX) [file pone.0346779.s005.docx]

**Table S4: Search terms used for OVID Medline and OVID Embase.**

| OVID Medline/ OVID Embase | 1. Impulsive Behavior/  2. self-control/  3. exp self control/ 4. "disruptive, impulse control, and conduct disorders"/ or gambling/  5. behavior control/ or social control, informal/  6. exp Inhibition, Psychological/ 7. impuls*.tw,kf.  8. disinhibit*.tw,kf.  9. inhibit*.tw,kf.  10. (state adj2 (impuls* or self-control or control or disinhibit* or inhibit*)).tw,kf.  11. (moment* adj2 (impuls* or self-control or control or disinhibit* or inhibit*)).tw,kf.  12. (daily adj2 (impuls* or self-control or control or disinhibit* or inhibit*)).tw,kf.  13. lack of control.tw,kf.  14. Delay Discounting/  15. lack of planning.tw,kf.  16. urgency.tw,kf.  17. perseverance.tw,kf.  18. premeditation.tw,kf.  19. non-planning.tw,kf.  20. sensation-seeking.tw,kf.  21. sensation seeking.tw,kf.  22. impulse control disorders/ or impulsiveness/  23. choice behavior/  24. exp Self-Regulation/  25. or/1-24  26. alcohol drinking/ or binge drinking/ or alcohol drinking in college/ or underage drinking/  27. Alcoholic Intoxication/  28. alcohol intoxication.tw,kf. 29. Alcoholism/  30. (alcohol* adj2 (abuse* or addict* or dependen* or disorder* or misuse)).tw,kf.  31. "marijuana use"/ or marijuana smoking/  32. mari#uana.tw,kf.  33. (mari#uana* adj2 (abuse* or addict* or dependen* or disorder* or misuse)).tw,kf.  34. (cannabis adj2 (abuse* or addict* or dependen* or disorder* or misuse)).tw,kf.  35. (cannabinoid* adj2 (abuse* or addict* or dependen* or disorder* or misuse)).tw,kf.  36. "tobacco use"/ or tobacco smoking/  37. smoking/ or pipe smoking/ or smoking reduction/ or smoking, non-tobacco products/ or tobacco smoking/ or vaping/  38. (tobacco* adj2 (abuse* or addict* or dependen* or disorder* or misuse)).tw,kf.  39. (nicotine adj2 (abuse* or addict* or dependen* or disorder* or misuse)).tw,kf.  40. Electronic Nicotine Delivery System  41. cannabis.tw,kf.  42. mari#uana.tw,kf.  43. tobacco.tw,kf.  44. nicotine.tw,kf.  45. "Cannabis Use"/ or exp Marijuana/ or exp Cannabis/  46. exp Smokeless Tobacco/ or exp Electronic Cigarettes/  47. "Tobacco Use Disorder"/ or "Alcohol Use Disorder"/ or "Cannabis Use Disorder"/  48. substance-related disorders/ or alcohol-related disorders/ or cannabis-related disorders/ or marijuana abuse/ or substance abuse, oral/ or "tobacco use disorder"/  49. Nicotine/  50. alcohol.tw,kf.  51. or/26-50  52. Ecological Momentary Assessment/  53. experience sampl*.tw,kf. 54. ecological momentary.tw,kf.  55. daily diary.tw,kf.  56. ambulatory assessment.tw,kf.  57. or/52-56  58. 25 and 51 and 57  59. exp animals/ not humans  60. 58 not 59 |
| --- | --- |

This table presents the complete Boolean search strings used to identify relevant studies in OVID Embase and OVID Medline.
