# Supplementary material for: State impulsivity and substance use: A systematic review and meta-analysis protocol
Source: PLoS One. 2026 Apr 7;21(4):e0346779. doi: 10.1371/journal.pone.0346779 (PMC13056172; doi:10.1371/journal.pone.0346779)
Supplement: S5 Table — This table presents the complete Boolean search strings used to identify relevant studies in OVID PsycINFO. (DOCX) [file pone.0346779.s006.docx]

**Table S5: Search terms used for OVID PsycInfo.**

| OVID PsycInfo | 1. exp Impulsiveness/  2. self-control/  3. choice behavior/  4. exp Self-Regulation/ 5. "disruptive, impulse control, and conduct disorders"/ or gambling/ 6. behavior control/ or social control, informal/  7. exp Behavioral Inhibition System/ or exp Behavioral Inhibition/ or exp Response Inhibition/  8. impuls*.tw.  9. disinhibit*.tw.  10. inhibit*.tw.  11. (state adj2 (impuls* or self-control or control or disinhibit* or inhibit*)).tw. 12. (moment* adj2 (impuls* or self-control or control or disinhibit* or inhibit*)).tw.  13. (daily adj2 (impuls* or self-control or control or disinhibit* or inhibit*)).tw. 14. lack of control.tw.  15. Delay Discounting/  16. lack of planning.tw.  17. urgency.tw.  18. premeditation.tw.  19. non-planning.tw.  20. sensation-seeking.tw.  21. sensation seeking.tw.  22. impulse control disorders/ or impulsiveness/  23. perseverance.tw.  24. or/1-23  25. alcohol drinking/ or binge drinking/ or alcohol drinking in college/ or underage drinking/ 26. Alcoholic Intoxication/  27. exp Alcohol Intoxication/  28. exp "Alcohol Use"/  29. Alcoholism/ 30. (alcohol* adj2 (abuse* or addict* or dependen* or disorder* or misuse)).tw. 31. "marijuana use"/ or marijuana smoking/  32. "Cannabis Use"/ or exp Marijuana/ or exp Cannabis/  33. mari#uana.tw.  34.  (mari#uana* adj2 (abuse* or addict* or dependen* or disorder* or misuse)).tw.  35. (cannabis adj2 (abuse* or addict* or dependen* or disorder* or misuse)).tw.  36. (cannabinoid* adj2 (abuse* or addict* or dependen* or disorder* or misuse)).tw.  37. "tobacco use"/ or exp Tobacco Smoking/  38. exp Smokeless Tobacco/ or exp Electronic Cigarettes/  39. "Tobacco Use Disorder"/ or "Alcohol Use Disorder"/ or "Cannabis Use Disorder"/  40. smoking/ or pipe smoking/ or smoking reduction/ or smoking, non-tobacco products/ or tobacco smoking/ or vaping/  41. (tobacco* adj2 (abuse* or addict* or dependen* or disorder* or misuse)).tw.  42. Nicotine/  43. (nicotine adj2 (abuse* or addict* or dependen* or disorder* or misuse)).tw.  44. substance-related disorders/ or alcohol-related disorders/ or cannabis-related disorders/ or marijuana abuse/ or substance abuse, oral/ or "tobacco use disorder"/  45. alcohol.tw.  46. cannabis.tw.  47. mari#uana.tw.  48. tobacco.tw.  49. nicotine.tw.  50. or/25-49  51. Ecological Momentary Assessment/  52. experience sampl*.tw.  53. daily diary.tw.  54. ambulatory assessment.tw.  55. or/51-54  56. exp animals/ not humans/  57. 24 and 50 and 55  58. 57 not 56 |
| --- | --- |

This table presents the complete Boolean search strings used to identify relevant studies in OVID PsycINFO.
